# 3D Face Recognition Based on Multiple Keypoint Descriptors and Sparse Representation

**DOI:** 10.1371/journal.pone.0100120

**Published:** 2014-06-18

**Authors:** Lin Zhang, Zhixuan Ding, Hongyu Li, Ying Shen, Jianwei Lu

**Affiliations:** 1 School of Software Engineering, Tongji University, Shanghai, China; 2 The Advanced Institute of Translational Medicine, Tongji University, Shanghai, China; University of Michigan, United States of America

## Abstract

Recent years have witnessed a growing interest in developing methods for 3D face recognition. However, 3D scans often suffer from the problems of missing parts, large facial expressions, and occlusions. To be useful in real-world applications, a 3D face recognition approach should be able to handle these challenges. In this paper, we propose a novel general approach to deal with the 3D face recognition problem by making use of multiple keypoint descriptors (MKD) and the sparse representation-based classification (SRC). We call the proposed method 3DMKDSRC for short. Specifically, with 3DMKDSRC, each 3D face scan is represented as a set of descriptor vectors extracted from keypoints by meshSIFT. Descriptor vectors of gallery samples form the gallery dictionary. Given a probe 3D face scan, its descriptors are extracted at first and then its identity can be determined by using a multitask SRC. The proposed 3DMKDSRC approach does not require the pre-alignment between two face scans and is quite robust to the problems of missing data, occlusions and expressions. Its superiority over the other leading 3D face recognition schemes has been corroborated by extensive experiments conducted on three benchmark databases, Bosphorus, GavabDB, and FRGC2.0. The Matlab source code for 3DMKDSRC and the related evaluation results are publicly available at http://sse.tongji.edu.cn/linzhang/3dmkdsrcface/3dmkdsrc.htm.

## Introduction

Recognizing the identity of a person with high confidence is a critical issue in various applications, such as e-banking, access control, passenger clearance, national ID card, etc. The need for reliable user authentication techniques has significantly increased in the wake of heightened concerns about security, and rapid advancement in networking, communication and mobility [1]. Biometrics, which refers to automatic identification of individuals based on their measurable physiological or behavioral attributes, is of great interest and has received considerable attention because of their high accuracy and convenience to use in the modern e-world. Due to the natural and non-intrusive nature of data acquisition, the face has many benefits when compared to other biometric identifiers.

Face recognition has received substantial attention over the last three decades. To date, the majority of implemented face recognition systems are based on 2D images. Unfortunately, despite the great efforts made over the last decades, face recognition using 2D images is still a great challenge due to kinds of adverse factors, such as illumination variation, pose changes, makeup, or facial expressions. The emergence of reliable and inexpensive 3D scanners has provided new opportunities for researchers to use 3D shape information of the face to obtain better performance [2]. 3D scanning has a major advantage over 2D imaging in that those nuisance factors have a relatively smaller influence. The 3D face recognition algorithms identify faces from the 3D shape of a person’s face. In the literature, some works in this field attempt to integrate discriminating information from 2D and 3D modalities simultaneously [3] and others depend solely on 3D information. In this paper, our discussions are confined only to the latter ones.

### Previous Work

The task of recognizing 3D face scans have been approached in various ways, leading to varying level of successes. Some representative and prominent works will be briefly reviewed here. The existing 3D face recognition algorithms can be roughly classified into “holistic-based” and “local-based” techniques.

The holistic techniques employ information from the whole face or at least from large regions of the 3D face. Many early-stage 3D face recognition algorithms were simply extended versions of holistic 2D approaches, in which the portrait images are replaced by range images. Typically, the input range images are aligned and then reformatted into feature vectors. After that, some statistical dimensionality reduction techniques, such as the principal component analysis (PCA) [4]–[8], the linear discriminant analysis (LDA) [9], [10], and the independent component analysis (ICA) [11], are adopted to learn the subspace of the feature vectors. Thereafter, facial images are projected onto the learned subspace and then are compared by means of a suitable metric in that space.

Apart from the aforementioned appearance-based schemes, there are also some other kinds of holistic techniques. Some researchers attempted to deal with the 3D face recognition problem by using “surface matching” techniques, in which the two facial surfaces under comparison are iteratively registered as closely as possible in 3D space by minimizing a distance metric. Representative examples belonging to this category are iterative closest point (ICP) and its various variants [12]–[15]. The ICP-based surface matching techniques are robust to variable facial poses and illumination variations. However, ICP-based registration procedures are not guaranteed to converge to a global minimum and they are computationally expensive. Another limitation of these methods is their sensitivity to facial expressions, which actually are non-rigid deformations of the facial surface [14]. Other methods rely on deforming facial surfaces into one another under some criteria, and use quantifications of these deformations as metrics for face comparison. Representative works belonging to this category include [16]–[18], in which elastic registration with morphable models were used. In order to deal with variable facial expressions, some researchers utilize geodesic distances between points on facial surfaces to define features that are eventually used for comparison. For the methods belonging to this category, they assume that geodesic distances are relatively invariant to small changes in facial expressions and can consequently help generate features that are robust to facial expressions. Motivated by these insights, Bronstein *et al*. [19], [20] proposed a 3D face recognition approach by matching intrinsic representations of facial features that are computed using multi-dimensional scaling. Samir *et al*. [21] proposed to use the level curves of the surface distance from the tip of nose as features for face recognition. Berretti *et al*. [22] used surface distances to extract equal-width iso-geodesic facial stripes, which in turn, were used as nodes in a graph-based recognition algorithm. However, approaches as proposed in [21], [22] are not able to deal with the problems caused by missing data or occlusions, since under these cases the shape of the level curves will definitely be affected. In [23], Mahoor and Abdel-Mottaleb represent each range image by ridge lines on the 3D surface of the face using a 3D binary image, namely ridge image, which is the locus of the points which have principal curvatures grater than a threshold. With respect to the matching strategy, they also resorted to ICP. The limitation of [23] lies in that it can only deal with frontal or near-frontal range scans. In [24], Drira *et al*. represent facial surfaces by radial curves emanating from the nose tips and use elastic shape analysis of those curves to develop a Riemannian framework for analyzing shapes of full facial surfaces. In [25], 3D face scans are represented in a canonical representation, namely, spherical depth map, from which spherical harmonic features can be derived. Smeets *et al*. in [26] proposed a geodesic distance matrix (GDM)-based representation scheme, in which the vector of eigenvalues of GDM was used as an isometry-invariant shape representation. Such a method is also sensitive to the problems aroused by missing data or occlusions.

Although 3D data can offer several great advantages over their 2D counterparts, the non-rigid deformations due to facial expressions, missing data, and self-occlusion problems caused in data acquisition severely affect the accuracy of 3D face recognition. To cope with these issues, another common framework is based on matching only parts or regions rather than matching full faces. In [27], Lee *et al*. extracted eight fiducial points that are geometrically invariant and then they used ratios of distances and angles between fiducial points as features, followed by an SVM classifier. Motivated by the research fruit of facial anthropometry, Gupta *et al*. proposed a 3D face recognition approach, namely “Anthroface 3D” [28]. In “Anthroface 3D”, ten anthropometric facial fiducial points are detected at first, and then the facial 3D Euclidean and geodesic distances between the detected fiducial points are employed as features. The weakness of such an approach is its sensitivity to the problems of missing data or occlusions as under these adverse conditions, it is nontrivial to faithfully detect the anthropometric fiducial points. In [29], Li *et al*. designed a feature pooling and ranking scheme to collect various types of low-level geometric features, such as the curvature at the vertex, the area of each triangle, and the length of each edge, and rank them according to their sensitivity to facial expressions. In [30], Faltemier *et al*. proposed a region ensemble based 3D face recognition framework. In their method, the nose tip is automatically selected and then 28 face regions around the face are extracted. When matching a gallery-probe pair, corresponding regions are matched at first using ICP and then the overall matching score is obtained as the fusion of the local matching results. Such an idea of part-based matching [30] was also explored in some other works, such as [31]–[36]. In [37], Elaiwat *et al*. explored the curvelet transform to detect salient points on the face scan and to build multi-scale local surface descriptors. Inspired by SIFT [38], which is a quite successful method for matching 2D images, Smeets *et al*. [39] developed a meshSIFT algorithm which could detect keypoints and build local descriptors for 3D meshes. Such an algorithm has been applied to 3D face recognition and promising results were reported on Bosphorus database [40].

### Overview of Our Approach

When missing data, large facial expressions, or occlusions exist in 3D face scans, it would be difficult for an approach based on holistic representations to succeed. Instead, methods resorting to local representations seem more appealing. For most state-of-the-art local representation based methods, it is imperative to detect some semantic fiducial points at first, such as the nose tip, the eye corners, the mouth corners, etc. However, it is nontrivial to design an approach that can automatically and robustly detect fiducial points when missing data, self-occlusions, or large expressions exist in face scans.

In this paper, we propose a novel general 3D face recognition scheme based on local representations. In such an approach, we require neither the alignment of facial range images nor the detection of meaningful fiducial points. Our approach is highly motivated by the success of a recent work designed for 2D partial face matching, namely MKDSRC (*M*ultiple *K*eypoint *D*escriptors and *S*parse *R*epresentation based *C*lassification) [41]. MKDSRC proposed by Liao *et al*. [41] is an alignment-free 2D partial face matching approach, in which each face is represented by a set of descriptor vectors extracted from keypoints and a multi-task SRC is used for classification. Such a method can address the problem of 2D partial face matching pretty well.

Specifically in our approach, for each 3D face scan F, we at first use meshSIFT [39] to extract from it multiple keypoints and then build the associated local descriptors. By using meshSIFT, keypoints are detected as mean curvature extrema in the scale space. The set of local descriptors derived from F; can be used as a representation of F. In order to build the gallery dictionary, all the local descriptors extracted from gallery samples are concatenated together. Given a probe face scan, its local descriptors are extracted at first and then its identity can be determined by using a multi-task SRC. The proposed method is called 3DMKDSRC (*3D M*ultiple *K*eypoint *D*escriptors and *S*parse *R*epresentation based *C*lassification). 3DMKDSRC uses a variable-sized description and accordingly each face scan is represented by a set of descriptors. Since the MKD dictionary comprises a large number of gallery descriptors, it is highly possible to sparsely represent descriptors from a probe scan, irrespective of whether it is a holistic, partial, or occluded one. 3DMKDSRC is particularly appropriate for matching 3D scans with missing parts, facial expressions, or occlusions. Its efficacy has been validated on three widely used benchmark databases.

The rest of this paper is organized as follows. Section 2 briefly reviews meshSIFT, based on which we extract from 3D face scans interest points and construct local descriptors. Section 3 presents our 3DMKDSRC approach in details. Section 4 reports the experimental results while Section 5 concludes the paper.

## meshSIFT

In our 3DMKDSRC approach, each 3D face scan is represented by a set of local descriptors extracted from keypoints. With respect to the scheme for keypoint detection and local descriptor construction for 3D scans, we resort to meshSIFT [39], which is an effective method designed for these particular tasks proposed quite recently. MeshSIFT was highly motivated by SIFT [38], which is now a widely used method to build scale invariant local descriptors for 2D gray-scale images. In this section, we briefly review the key steps of meshSIFT.

### 1. Keypoint Detection

**Figure 1:**
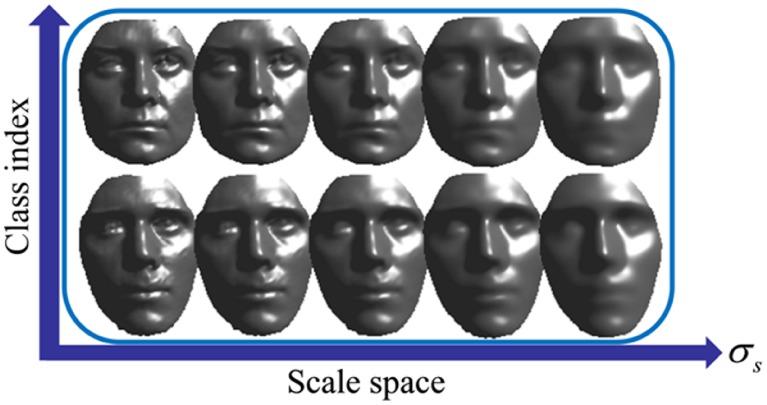
Shapes of two face scans in the scale space.

The keypoint detection step in meshSIFT is similar to SIFT. A scale space containing smoothed versions of the input mesh is constructed at first as:
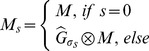
(1)where *M* stands for the original mesh, 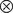 stands for the convolution operation, and 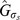 stands for the approximated Gaussian filter with scale *σ_s_*. These scales {*σ_s_*} vary exponentially as *σ_s_*  = 2*^s/k^σ*_0_, where *k* stands for the number of total scales. Since the number of convolutions is discrete, *σ_s_* is approximated as:
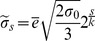
(2)with 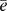 the average edge length. Fig. 1 shows the shapes of two face scans in the scale space.

**Figure 2:**
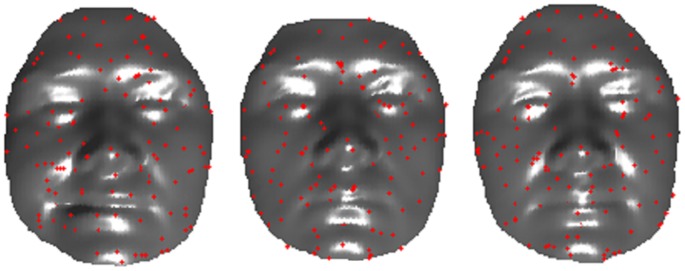
Keypoint detection results on three face scans of the same face.

To detect keypoints in the scale space, the mean curvature is computed for each vertex *i* at each scale *s* as:
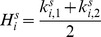
(3)where 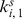 and 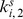 respectively stand for the maximum and minimum curvatures for each vertex *i* at scale *s*. The difference between subsequent scales could be computed as:



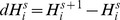
(4)A vertex is selected as a keypoint only when its value 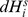 is larger or smaller than all its neighboring vertices in all upper, current, and lower scales. The scale *σ_s_* at which the extremum is obtained is assigned to each keypoint. Fig. 2 shows an example of keypoint detection results of 3 face scans collected from the same person.

### 2. Local Descriptor

Having detected keypoints, the next step is to describe them with local descriptors which actually summarize the local neighborhood information around them. In order to obtain an orientation-invariant descriptor, each keypoint is assigned a canonical orientation. With such a canonical orientation, it is possible to construct a local reference frame in which the vertices of the neighborhood can be expressed independent of the facial pose.

For a keypoint *P*, all vertices within a spherical region of radius 9*σ_s_* around it are its neighboring points. For each neighboring point, its normal vector is computed and its geodesic distance to *P* is determined based on the fast marching algorithm [42]. The normal vectors of these points are projected onto the tangent plane of the mesh containing *P*. The projected normal vectors are gathered in a weighted histogram with 360 bins. Each histogram entry is Gaussian weighted with the geodesic distances to *P*. The highest peak in the histogram and the peaks above 80% of this highest peak value are selected as canonical orientations. For a keypoint which has more than one canonical orientations, it can be regarded as multiple keypoints, each assigned one of the canonical orientations.

**Figure 3:**
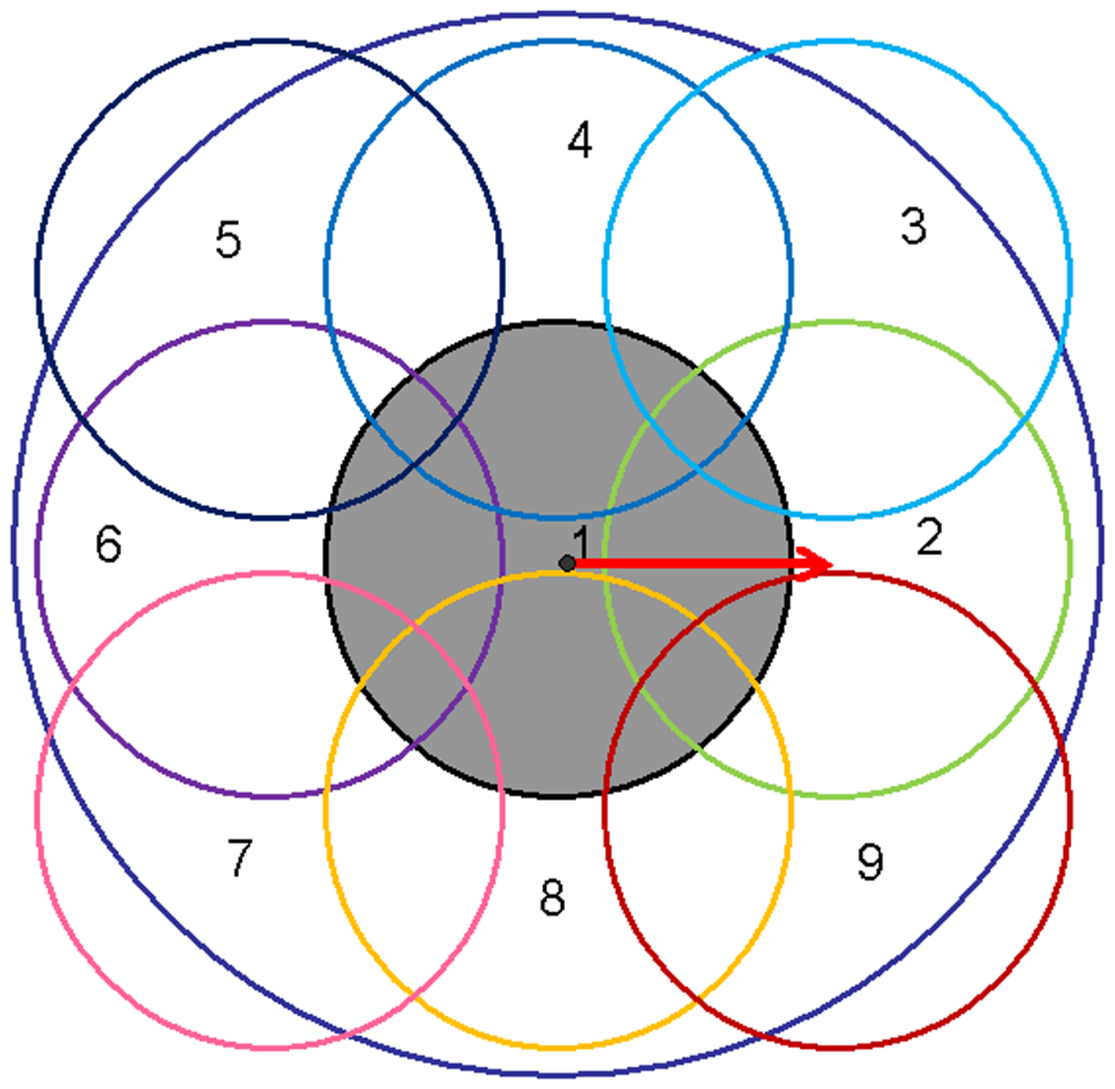
Nine regions involved in the computation of the local descriptor. The red arrow indicates the canonical orientation.

The generation of a local descriptor for *P* is based on 9 sub-regions. As described in Fig. 3, the locations of these 9 regions are based on the canonical orientation of *P*. The geodesic distances from the centers of regions 2, 4, 6 and 8 to *P* are all 4.5 *σ_s_*, while the geodesic distances from the centers of regions 3, 5, 7 and 9 to *P* are all 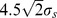.

For each of the 9 regions, two histograms *p_S_* and *p_θ_* are used for generating the descriptor. The first histogram contains the shape index which is expressed as:
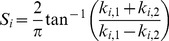
(5)where *k_i_*_,1_ and *k_i_*_,2_ are the maximum and the minimum curvatures, respectively. The second histogram contains the slant angles, which are defined as the angles between the projected normals and the canonical orientation. Both the shape index and the slant angle histograms are Gaussian weighted with the geodesic distances to *P*. Finally, the histograms are concatenated in a vector form as ***f*** = [*p_S_*_,1_ *p_θ_*_,1_…*p_S_*_,9_ *p_θ_*_,9_]*^T^* and ***f*** is regarded as the descriptor of *P*. Consequently, each 3D face scan can be represented as a set of descriptor vectors **F** = [***f***_1_,…, ***f*** *_n_*], where each ***f*** *_i_* is a local descriptor vector.

## 3DMKDSRC

In this section, the proposed 3D face recognition scheme 3DMKDSRC will be presented in details.

### 1. Construction of the Gallery Dictionary

For each sample 3D face scan in the gallery set, its local descriptors could be computed by meshSIFT. Then, the gallery dictionary is constructed by concatenating these descriptors together. Suppose that there are *C* subjects in gallery and for each subject *i* there are totally *n_i_* derived descriptors. Usually, these *n_i_* descriptors are obtained from multiple samples of the subject *i*. For the *i* ^th^ subject, let
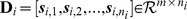
(6)


where *m* here stands for the descriptor dimension. The gallery dictionary **D** can be simply constructed by concatenating these **D** *_i_*s as:
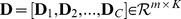
(7)where *K* here represents the total number of descriptors in the gallery set. Typically, *K* is very large, making **D** an over-complete description space of the *C* classes. According to the theory of compressed sensing, a sparse solution is possible for an over-complete dictionary [43]; therefore, any descriptor from a probe face scan can be expressed by a sparse linear combination of the items from the dictionary **D**.

### 2. Multi-task Sparse Representation

**Figure 4:**
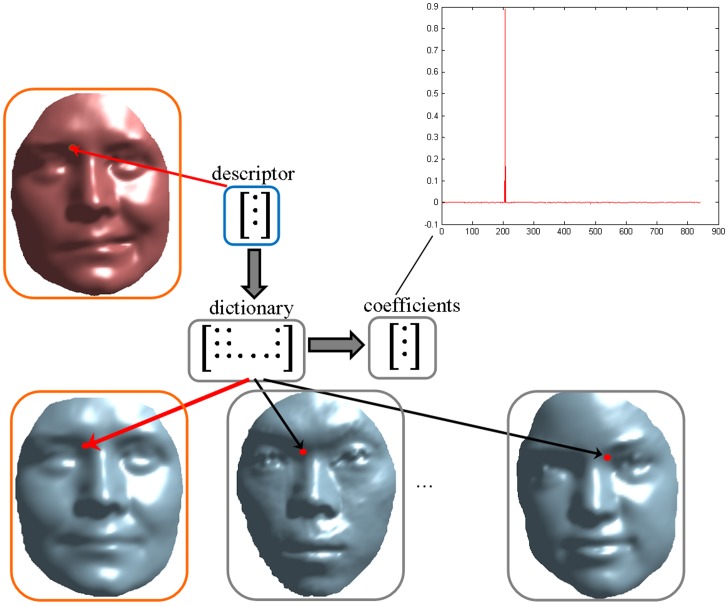
If a query descriptor has a matched descriptor in gallery, the coefficient would be very sparse.

Given a probe 3D face scan, we at first compute from it a set of local descriptors:
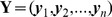
(8)with *n* the number of keypoints detected from this scan. Then, the sparse representation problem is formulated as:
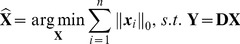
(9)where 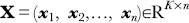 is the sparse coefficient matrix, and ||·||_0_ denotes the *l*_0_-norm of a vector. However, the solution to this problem is NP-hard. As suggested by the research results of compressed sensing [44], sparse signals can be well recovered with a high probability via the *l*_1_-minimization. Therefore, Eq. (9) can be approximated by:
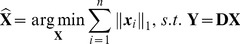
(10)where ||*·*||_1_ represents the *l*_1_-norm of the vector. This is a multi-task problem as both **X** and **Y** have multiple columns. Equivalently, we can solve the following set of *n l*_1_-minimization problems, one for each probe descriptor ***y*** *_i_*:



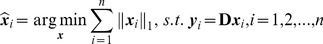
(11)To solve Eq. (11), several prominent algorithms have been developed in the past few years, including Homotopy [45], FISTA [46], DALM [47], SpaRSA [48], *l*_1__*ls* [49], etc. In our implementation, we use the Homotopy algorithm proposed in [45]. Usually, if the identity of the probe face scan is covered by the gallery set, the coefficient vectors of its local descriptors would be very sparse as illustrated in Fig. 4.

Inspired by [41], [50], we adopt the following multi-task SRC to determine the identity of the probe face scan:
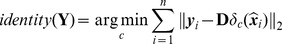
(12)where *δ_c_*(·) is a function which selects only the coefficients corresponding to class *c*. Eq. (12) makes use of the sum of reconstruction residuals of the *n* descriptors with respect to each class to determine the identity of the input face scan.

### 3. Dictionary Shrinking and Sparsity Criterion

In practice, the size (*K*) of the dictionary can be extremely large, making it difficult to solve Eq. (11). Hence, we adopt a similar idea as Liao *et al*. [41] to derive a fast approximate solution. For each probe descriptor ***y*** *_i_*, we first compute:
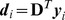
(13)


Then, for each ***y*** *_i_*, we only keep *L* (*L<<K*) descriptors in **D** according to the *L* largest values of ***d*** *_i_*, resulting in a small sub-dictionary 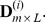 Then, **D** is replaced by **D** ^(*i*)^ in Eq. (11) and Eq. (12) is adjusted accordingly. In our implementation, *L* is set to 400.

In addition, we assume that if the identity of the probe face scan belongs to the *j^th^* subject of the gallery, the entries of 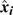 should be small except those associated with the *j^th^* subject. If the coefficients 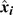 are not concentrated on any subject and instead values of 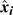 spread evenly over all the gallery subjects, ***y*** *_i_* is likely to be a noisy descriptor and it can provide little discriminative information. Thus, such 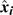 will not be considered when computing Eq. (12).

**Figure 5:**
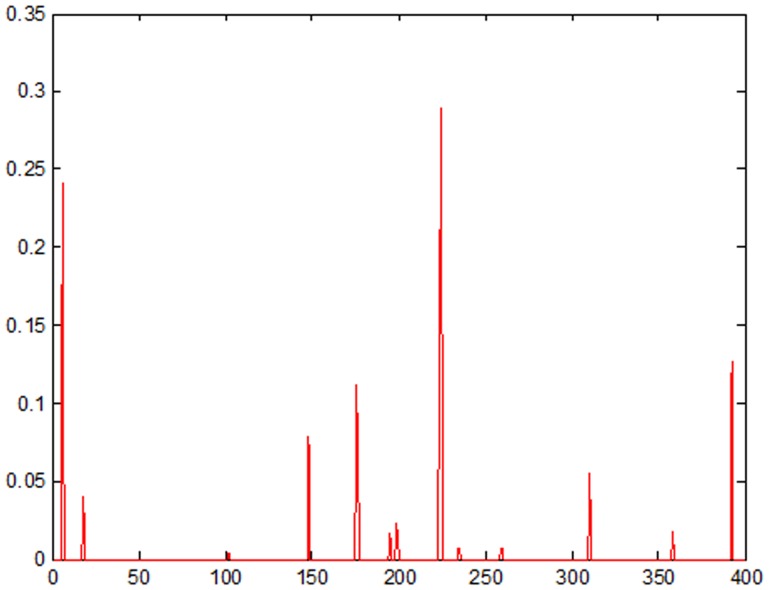
An example of a coefficient vector which is not sparse.

To evaluate the sparsity of 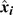, we use,
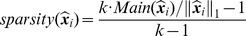
(14)where *k* is the number of subjects in **D** ^(*i*)^ and 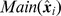 stands for the summation of absolute values of coefficients in 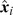 corresponding to the first 5 percent of subjects with higher sums of absolute coefficients. If 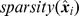 is larger than a threshold (0.8 in our implementation), we consider that 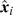 is sparse enough and it will be involved in the further determination of identity (see Eq. (12)). Fig. 5 shows an example of the distribution of a coefficient vector which is not sparse.

**Figure 6:**
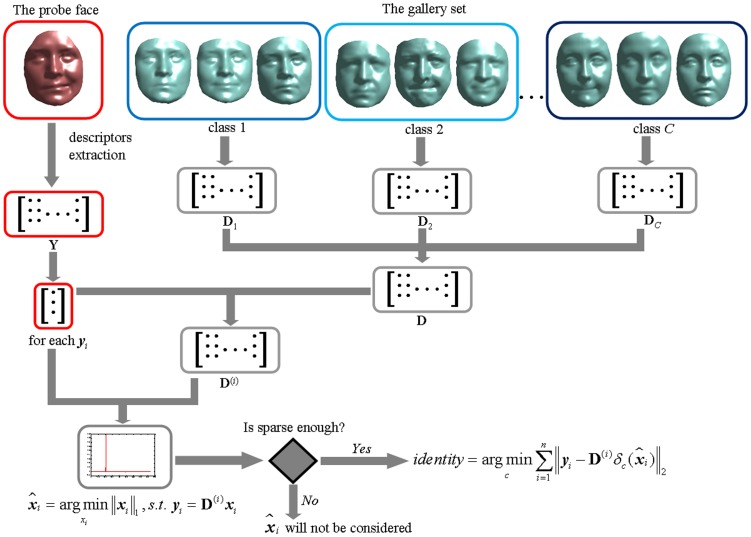
The overall flowchart of 3DMKDSRC.

The overall pipeline of our proposed 3DMKDSRC algorithm is illustrated in Fig. 6.

## Experimental Results and Discussions

In this section, we will provide a comparative performance analysis of our method with the other state-of-the-art or representative approaches using three public datasets, Bosphorus, GavabDB, and FRGC2.0.

### 1. Experiments on Bosphorus

The Bosphorus database [40] consists of 4666 facial range scans from 105 different subjects and is acquired by an Inspeck Mega Capturor 3D scanner leading to 3D point clouds of approximately 35000 points. In Bosphorus, facial expression variations, pose variations, and occlusions are present. The majority of the subjects are aged between 25 and 35.

In our experiment, we chose 3 face scans with neutral expressions to form the gallery set, making the gallery set have 315 samples. When forming the test set, two cases were considered. In the first case, the test set included all the remaining samples, while in the second case the test set only contained remaining frontal samples. Besides 3DMKDSRC, meshSIFT was also evaluated under the same experimental settings. The identification results in terms of rank-1 recognition rate are summarized in Table 1. In addition, results of several other algorithms are also reported. They include ICP based method [6], PCA based method [6], Alyuz *et al*.’s method [32], Dibekliglu *et al*.’s method [33], and Hajati *et al*.’s method [34], It needs to be noted that experiments conducted in [6], [32] and [33] were based on Bosphorus 2.0 which contains 2491 facial scans collected from 47 subjects, smaller than the one used in our experiments. In addition, only frontal samples were involved in those experiments.

**Table 1:** Rank-1 recognition rates on Bosphorus.

Approach	Size of gallery set	Size of probe set	Rank-1 RR
**3DMKDSRC (all)**	**315**	**4351**	**95.03%**
**3DMKDSRC (frontal)**	**315**	**3543**	**98.65%**
meshSIFT (all)	315	4351	92.99%
meshSIFT (frontal)	315	3543	96.56%
ICP [6] (frontal)	47	1508	72.4%
PCA [6] (frontal)	47	1508	70.6%
Alyuz *et al.* [32] (frontal)	47	1508	95.3%
Dibekliglu *et al*. [33] (frontal)	47	1527	89.2%
Hajati *et al*. [34] (all)	–	–	69.1%

From the results listed in Table 1, it can be seen that the proposed 3DMKDSRC performs much better than the other methods evaluated.

### 2. Experiments on GavabDB

GavabDB [51] is designed to be the most expression rich and noise prone 3D face database. The database consists of the Minolta Vi-700 laser range scans from 61 subjects. For each subject, 9 scans are collected, covering different poses and various facial expressions. We skipped those 2 types of scans which are largely rotated (±90 degrees). For each subject, we chose 3 neutral faces to build the gallery set. When forming the test set, two cases were considered. In the first case, the test set included all the remaining samples, while in the second case the test set only contained remaining neutral samples. Besides 3DMKDSRC, meshSIFT was also evaluated using the same experimental protocol. The rank-1 recognition rates are summarized in Table 2. In addition, results of several other representative algorithms, including Moreno *et al*.’s method [7], [35], Mousavi *et al*.’s method [8], and Mahoor *et al*.’s method [23], are also reported in Table 2 for comparison.

**Table 2:** Rank-1 recognition rates on GavabDB.

Approach	Size of gallery set	Size of probe set	Rank-1 RR
**3DMKDSRC (neutral)**	**183**	**61**	**100%**
**3DMKDSRC (all)**	**183**	**244**	**92.62%**
meshSIFT (neutral)	183	61	98.36%
meshSIFT (all)	183	244	86.22%
Moreno *et al*. [7] (all)	305	122	77.9%
Moreno *et al*. [35] (neutral)	60	60	78%
Mousavi *et al.* [8] (neutral)	61	61	91%
Mahoor *et al.* [23] (neutral)	61	183	95%

The superiority of 3DMKDSRC over the other competitors can be clearly observed from the results listed in Table 2. Particularly, when the test set only contains samples with neutral expressions, the rank-1 recognition rate of 3DMKDSRC can reach 100%, which is quite amazing.

### 3. Experiments on FRGC2.0

FRGC2.0 [52] database contains 4007 640×480 3D range scans which were taken under controlled illumination conditions by a Minolta Vivid 900/910 series 3D sensor. The face scans came from 466 different subjects.

In this experiment, we randomly chose 3 face scans for each subject to form the gallery set. For the subject which has less than 3 samples, we just put all its samples in the gallery. The rest of the faces in the database were used for testing. The rank-1 recognition rates obtained under those settings by 3DMKDSRC and meshSIFT are listed in Table 3. Actually, some state-of-the-art methods, such as [24], could achieve higher recognition accuracy than 3DMKDSRC on FRGC2.0. However, it should be noted that those methods would usually apply a complicated data preprocessing procedure (e.g., hole filling) on the face scans in FRGC2.0 to improve the data quality. By contrast, in our experiments, no extra data preprocessing was performed. That’s the main cause accounting for the lower recognition accuracies of 3DMKDSRC and meshSIFT reported here. 3D data preprocessing is an independent area and in the future we may try to give deeper investigations in this field.

**Table 3:** Rank-1 recognition rates on FRGC2.0.

Approach	Size of gallery set	Size of probe set	Rank-1 RR
**3DMKDSRC**	**1259**	**2748**	**89.29%**
meshSIFT	1259	2748	87.85%

## Conclusions

In this paper, we have addressed the problem of 3D face recognition and proposed a novel approach, namely 3DMKDSRC. 3DMKDSRC represents each 3D face scan by a set of keypoint descriptor vectors extracted by meshSIFT and constructs a large dictionary from all the gallery descriptors. At the testing stage, descriptors of a probe face scan can be sparsely represented by the dictionary, and its identity can be determined accordingly by solving a multi-task SRC problem. 3DMKDSRC is particular appropriate for matching range scans with missing parts, large expressions, or occlusions. Its efficacy has been corroborated by the extensive experiments conducted on various benchmark databases.

## References

[1] Jain AK, Flynn PJ, Ross A (2007) Handbook of Biometrics. Springer.

[2] Bowyer K, Chang K, Flynn P (2006) A survey of approaches and challenges in 3D and multi-modal 3D+2D face recognition. Computer Vision and Image Understanding 101: 1–15.

[3] Mian A, Bennamoun M, Owens R (2007) An efficient multimodal 2D-3D hybrid approach to automatic face recognition. IEEE Transactions on Pattern Analysis and Machine Intelligence 29: 1927–1943. 17848775 10.1109/TPAMI.2007.1105

[4] Chang KI, Bowyer KW, Flynn PJ (2005) An evaluation of multimodal 2D+3D face biometrics. IEEE Transactions on Pattern Analysis and Machine Intelligence 27: 619–624. 15794165 10.1109/TPAMI.2005.70

[5] Russ T, Boehnen C, Peters T (2006) 3D face recognition using 3D alignment for PCA. Proceedings of the CVPR, 1391–1398.

[6] Alyuz N, Gokberk B, Dibekliglu H, Savran A, Salah AA, et al (2008) 3D face recognition benchmarks on the Bosphorus database with focus on facial expressions. Proceedings of the BIOID, 1–7.

[7] Moreno AB, Sanchez A, Velez JF, Diaz FJ (2005) Face recognition using 3D local geometrical features: PCA vs SVM. Proceedings of the ISPA, 185–190.

[8] Mousavi MH, Faez K, Asghari A (2008) Three dimensional face recognition using SVM classifier. Proceedings of the ICIS, 208–213.

[9] Heseltine T, Pears N, Austin J (2004) Three-dimensional face recognition: A fishersurface approach. Proceedings of the ICIAR, 684–691.

[10] BenAbdelkader C, Griffin PA (2005) Comparing and combining depth and texture cues for face recognition. Image and Vision Computing 23: 339–352.

[11] Hesher C, Srivastava A, Erlebacher G (2003) A novel technique for face recognition using range imaging. Proceedings of the Seventh International Symposium on Signal Processing and Its Applications, 201–204.

[12] Besl P, McKay N (1992) A method for registration of 3D shapes. IEEE Transactions on Pattern Analysis and Machine Intelligence 14: 239–256.

[13] Russ TD, Koch MW, Little CQ (2005) A 2D range Hausdorff approach for 3D face recognition. Proceedings of the CVPR, 169–176.

[14] Lu X, Jain AK, Colbry D (2006) Matching 2.5D face scans to 3D models. IEEE Transactions on Pattern Analysis and Machine Intelligence 28: 31–43. 16402617 10.1109/TPAMI.2006.15

[15] Koudelka ML, Koch MW, Russ TD (2005) A prescreener for 3D face recognition using radial symmetry and the Hausdorff fraction. Proceedings of the CVPR Workshops.

[16] Kakadiaris I, Passalis G, Toderici G, Murtuza M, Lu Y, et al (2007) Three-dimensional face recognition in the presence of facial expressions: An annotated deformable model approach. IEEE Transactions on Pattern Analysis and Machine Intelligence 29: 640–649. 17299221 10.1109/TPAMI.2007.1017

[17] Lu X, Jain A (2008) Deformation modeling for robust 3D face matching. IEEE Transactions on Pattern Analysis and Machine Intelligence 30: 1346–1357. 18566490 10.1109/TPAMI.2007.70784

[18] Passalis G, Perakis P, Theoharis T, Kakadiaris I (2011) Using facial symmetry to handle pose variations in real-world 3D face recognition. IEEE Transactions on Pattern Analysis and Machine Intelligence 33: 1938–1951. 21383396 10.1109/TPAMI.2011.49

[19] Bronstein AM, Bronstein MM, Kimmel R (2005) Three-dimensional face recognition. International Journal of Computer Vision 64: 5–30.

[20] Bronstein AM, Bronstein MM, Kimmel R (2007) Expression-invariant representations of faces. IEEE Transactions on Image Processing 16: 188–197. 17283777 10.1109/tip.2006.884940

[21] Samir C, Srivastava A, Daoudi M, Klassen E (2009) An intrinsic framework for analysis of facial surfaces. International Journal of Computer Vision 82: 80–95.

[22] Berretti S, Bimbo AD, Pala P (2010) 3D face recognition using isogeodesic stripes. IEEE Transactions on Pattern Analysis and Machine Intelligence 32: 2162–2177. 20975115 10.1109/TPAMI.2010.43

[23] Mahoor MH, Abdel-Mottaleb M (2009) Face recognition based on 3D ridge images obtained from range data. Pattern Recognition 42: 445–451.

[24] Drira H, Amor BB, Srivastava A, Daoudi M, Slama R (2013) 3D face recognition under expressions, occlusions, and pose variations. IEEE Transactions on Pattern Analysis and Machine Intelligence 35: 2270–2283. 23868784 10.1109/TPAMI.2013.48

[25] Liu P, Wang Y, Huang D, Zhang Z, Chen L (2013) Learning the spherical harmonic features for 3-D face recognition. IEEE Transactions on Image Processing 22: 914–925. 23060332 10.1109/TIP.2012.2222897

[26] Smeets D, Hermans J, Vandermeulen D, Suetens P (2012) Isometric deformation invariant 3D shape recognition. Pattern Recognition 45: 2817–2831.

[27] Lee Y, Song H, Yang U, Shin H, Sohn K (2005) Local feature based 3D face recognition. Proceedings of the AVBPA, 909–918.

[28] Gupta S, Markey MK, Bovik AC (2010) Anthropometric 3D face recognition. International Journal of Computer Vision 90: 331–349.

[29] Li X, Jia T, Zhang H (2009) Expression-insensitive 3D face recognition using sparse representation. Proceedings of the CVPR, 2575–2582.

[30] Faltemier TC, Bowyer KW, Flynn PJ (2008) A region ensemble for 3-D face recognition. IEEE Transactions on Information Forensics and Security 3: 62–73.

[31] Spreeuwers L (2011) Fast and accurate 3D face recognition using registration to an intrinsic coordinate system and fusion of multiple region classifiers. International Journal of Computer Vision 93: 389–414.

[32] Alyuz N, Gokberk B, Akarun L (2008) A 3D face recognition system for expression and occlusion invariance. Proceedings of the BTAS, 1–7.

[33] Dibekliglu H, Gokberk B, Akarun L (2009) Nasal region based 3D face recognition under pose and expression variations. Proceedings of the ICB, 309–318.

[34] Hajati F, Raie AA, Gao Y (2012) 2.5D face recognition using patch geodesic moments. Pattern Recognition 45: 969–982.

[35] Moreno AB, Sanchez A, Velez JF, Diaz FJ (2003) Face recognition using 3D surface-extracted descriptors. Proceedings of the IMVIP.

[36] Alyuz N, Gokberk B, Akarun L (2013) 3-D face recognition under occlusion using masked projection. IEEE Transactions on Information Forensics and Security 8: 789–802.

[37] Elaiwat S, Bennamoun M, Boussaid F, El-Sallam A (2014) 3-D face recognition using curvelet local features. IEEE Signal Processing Letters 21: 172–175.

[38] Lowe DG (2004) Distinctive image feature from scale-invariant keypoints. International Journal of Computer Vision 60: 91–110.

[39] Smeets D, Keustermans J, Vandermeulen D, Suetens P (2013) MeshSIFT: Local surface features for 3D face recognition. Computer Vision and Image Understanding 117: 158–169.

[40] Savran A, Alyuz N, Dibekliglu H, Celiktutan O, Gokberk B, et al (2008) Bosphorus database for 3D face analysis. Proceedings of the Workshop on Biometrics and Identity Management, 47–56.

[41] Liao S, Jain AK, Li SZ (2013) Partial face recognition: An alignment-free approach. IEEE Transactions on Pattern Analysis and Machine Intelligence 35: 1193–1205. 23520259 10.1109/TPAMI.2012.191

[42] Kimmel R, Sethian JA (1998) Computing geodesic paths on manifolds. Proc. National Academy of Sciences 95: 8431–8435. 10.1073/pnas.95.15.8431 PMC21092 9671694

[43] Tibshirani R (1994) Regression shrinkage and selection via the Lasso. Journal of the Royal Statistical Society, Series B 58: 267–288.

[44] Donoho D (2006) For most large underdetermined systems of linear equations the minimal *l* _1_-norm solution is also the sparsest solution. Communications on Pure and Applied Mathematics 59: 797–829.

[45] Donoho D, Tsaig Y (2008) Fast solution of *l* _1_-norm minimization problem when the solution may be sparse. IEEE Transactions on Information Theory 55: 4789–4812.

[46] Beck A, Teboulle M (2009) A fast iterative shrinkage-thresholding algorithm for linear inverse problems. SIAM Journal on Imaging Sciences 2: 183–202.

[47] Yang J, Zhang Y (2011) Alternating direction algorithms for *l* _1_-problems in compressive sensing. SIAM Journal on Scientific Computing 33: 250–278.

[48] Wright SJ, Nowak RD, Figueiredo MAT (2009) Sparse reconstruction by separable approximation. IEEE Transactions on Signal Processing 57: 2479–2493.

[49] Kim SJ, Koh K, Lustig M, Boyd S, Gorinevsky D (2007) An interior-point method for large-scale *l* _1_-regularized least squares. IEEE Journal on Selected Topics in Signal Processing 1: 606–617.

[50] Wright J, Yang A, Ganesh A, Sastry S, Ma Y (2009) Robust face recognition via sparse representation. IEEE Transactions on Pattern Analysis and Machine Intelligence 31: 210–227. 19110489 10.1109/TPAMI.2008.79

[51] Moreno AB, Sánchez A (2004) GavabDB: A 3D face database. Available: http://gavab.escet.urjc.es. Accessed 2014 Apr 08.

[52] Phillips PJ, Flynn PJ, Scruggs T, Bowyer KW, Chang J, et al (2005) Overview of the face recognition grand challenge. Proceedings of the CVPR, 20–25.

